# A protocol for a scoping review to map the assessment approaches in optometry education programmes globally

**DOI:** 10.1186/s13643-022-01906-7

**Published:** 2022-02-21

**Authors:** Diane van Staden, Verusia Chetty, Alvin Jeffrey Munsamy

**Affiliations:** 1 Discipline of Optometry, 6th Floor, E Block, Westville campus, University Road, Westville, Durban, South Africa; 2 Discipline of Physiotherapy, Room E2-512, 5th Floor, E Block, Westville campus, University Road, Westville, Durban, South Africa; 3 Discipline of Optometry, Room E5-642, 6th Floor, E Block, Westville campus, University Road, Westville, Durban, South Africa

**Keywords:** Optometry education, Assessment approaches, Global optometry, Competency-based training, Eye health

## Abstract

**Background:**

The training of optometrists aims to prepare practitioners with critical thinking skills who utilise their education and experience to solve clinical problems in real-life practice. Professional competencies should inform assessment, and as such, assessment methods for learning should encompass a wide range of approaches. The objective of this scoping review is therefore to map assessment approaches utilised within optometry education programmes globally.

**Methods:**

This study is a scoping review based on the PRISMA methodology. The review will be guided by the following research question, “What are the assessment approaches that inform optometry training globally?”. This was validated by the Population-Concept-Context framework according to the methodology for Joanna Briggs Institution Scoping Reviews. Relevant peer-reviewed studies and grey literature conducted during the last 10 years will be identified from electronic databases including CINAHL, PubMed, PROquest and ERIC. The search strings using keywords such as “Optometry students and staff”, “Assessments” and “Optometry education” will be conducted using Boolean logic. An independent reviewer will conduct all title screening, two independent reviewers will conduct abstract and full article screening, followed by data extraction. Thereafter, a thematic analysis will be conducted. The Mixed Method Appraisal Tool version 2018 will be used for quality appraisal of mapped studies.

**Discussion:**

The review will document evidence of assessment approaches utilised in optometry training globally. Considering the exit level competencies required in the basic job function of an optometrist, a coherence in assessment approaches and relevant rationale for these would be expected, if the accredited (regulated) training programmes follow a competency-based model.

## Introduction

Optometry is a health science discipline which trains eye health professionals to varying scope of practice levels, dependent on country or region. Optometrists are primary health care providers who independently examine, diagnose and manage diseases and disorders of the eye and visual system, as well as diagnose related systemic conditions [1]. The training of optometrists therefore aims to prepare eye health practitioners with critical thinking skills who can utilise their education and experience to solve clinical problems in real-life practice.

Assessments have historically been used to measure the performance of students [2] and are a central part of teaching and learning in higher education. Assessment is considered a process of gathering information from multiple sources in order to develop an understanding of what students know, understand and can do with their knowledge as a result of their educational experiences [3]. Assessment is therefore central to professional development and a crucial step in the educational process.

In 2000, the Association of Schools and Colleges of Optometry in the USA defined ‘Attributes of Students Graduating from Schools and Colleges of Optometry’. These were reviewed in 2011 to represent contemporary thinking about the competencies required for new graduates of optometry programs as well as trends in health professions education and health care delivery systems. It recognised the need for expanding strategies which provide for ongoing scrutiny of individual and programmatic results and highlighted the imperative to make appropriate adjustments to programme outcomes and techniques used to teach or develop professional competencies [4]. Globally, there is a demand for highly qualified health professionals. Professional competencies should therefore inform assessment, and as such, assessment methods for learning should encompass a wide range of approaches [5]. Fundamentally, assessment approaches should have the capacity to accurately evaluate competencies, skills and attitudes acquired during training of health professionals [6]. Therefore, before making a choice of assessment method, important questions should be asked by educators i.e. what should be assessed, why that particular aspect needs to assessed and what the best method of assessment should be to achieve the intended learning outcome/s. As such, education programmes should ensure that assessment approaches are comprehensive and robust enough to assess required attributes along with testing for essential knowledge and skills [7].

Not much is known about assessment methods utilised in optometry education programmes globally. Since what is assessed and the type of assessment approach utilised plays a significant role in what is learnt [6], it is important to gain an understanding of what assessment approaches are being utilised in optometry education programmes globally. The objective of this scoping review is therefore to explore assessment approaches utilised within accredited optometry education programmes globally (regulated by relevant regional bodies), in order to gain an understanding of similarities and/or differences in approaches, and the possible rationale for these in light of expected competencies and desired graduate attributes. The review will include all studies published in English between 2009 and 2019 relating to accredited professional optometry training programmes globally.

## Methodology

### Objective

The objective of the study is to map available evidence of assessment approaches in optometry education programmes globally.

### Identifying the research question

What are the assessment approaches that inform optometry training globally?

### Eligibility of research question

The study will use a Population-Concept-Context framework to determine eligibility of the primary research question as shown in Table 1 below [8].

**Table 1:** Framework for determining eligibility of research question

P-Population	Optometry students and staff
C-Concept	Assessments used in optometry training
C-Context	Optometry education programmes

### Identify relevant studies

Included in this study will be primary research articles, published in peer-reviewed journals, as well as grey literature. The following electronic databases will be used: CINAHL, PubMed, PROquest and ERIC. The search terms will include “optometry students and/or staff” “assessment,” “optometry education programme/s,” Boolean terms, “AND”; “OR”; “NOT” will be used to separate keywords. A pilot search using the above keywords to determine the feasibility of the study was conducted. The search strategy will be adapted to each database. Each search will be documented showing details such as the keywords, date of search, search engine and the number of publications retrieved. An example of the results of a pilot searches have been included in the Table 2 below.

**Table 2:** Results of a pilot search

Keywords searched	Date of search	Data base	Number of publications retrieved
(((((((((optometry teachers OR optometry students OR optometry faculty OR optometry educators)) AND (assessment OR tests OR assignment OR “OSCE” OR “OSPE” OR summative OR formative OR competency-based OR “optometry board exam*”)) AND (school OR training program* OR optometry curriculum OR university OR college OR education )) AND “last 10 years”[PDat] AND Humans [Mesh])) AND “last 10 years”[PDat] AND Humans[Mesh])) AND “last 10 years”[PDat] AND Humans[Mesh]) Filters: Humans	4/09/19	Pubmed	180

### Study selection

#### Eligibility criteria

Led by the study research question, the researchers created the inclusion/exclusion criteria to achieve accurate detection and selection of appropriate studies.

##### Inclusion criteria

The following data sources will be included:

- Primary research studies published between 2009 and 2019 (in the past 10 years)
- Grey literature sourced from websites of schools of optometry, universities, regulatory bodies, government educational departments, etc.
- Studies reporting evidence on the assessment approaches used in optometry education programmes

Studies in all languages

##### Exclusion criteria

We will exclude studies that contain the following:

- Evidence from non-regulated optometry education programmes
- Evidence on optometry education that does not-encompass assessment approaches
- Continuing development education programmes for established graduate professionals
- Eye health training programmes other than optometry

#### Charting the data

The data charting form as shown in Table 3 has been constructed to suit the context of this study. All data will be charted independently by two members of the team, which may be subject to change should the reviewers in consultation decide valuable data is omitted. The chart in Table 3 contains study metrics (author, publication date, etc.), population characteristics and study aims and outcomes. All extracted data from this chart will be thematically analysed either quantitatively or qualitatively to best facilitate answering of the research question. All coding will be done by two members of the team to minimise systematic bias or potential errors.

**Table 3:** Data charting form

**Author and year**	**Geographic location, Study setting**	**Population student/educator**	**Aim**	**Study design**	**Sample size**	**Outcome measure**	**Key findings**	**Conclusion**

### Collating, summarising and reporting

The primary objective of this study is to identify evidence on the assessment approaches used in optometry programmes globally from the charted data so as to map the assessment practices across various schools, education programs or regions. The framework method of analysis will be used to deductively analyse the data using competency-based education as a guiding framework for the analysis process.

**Fig. 1:**
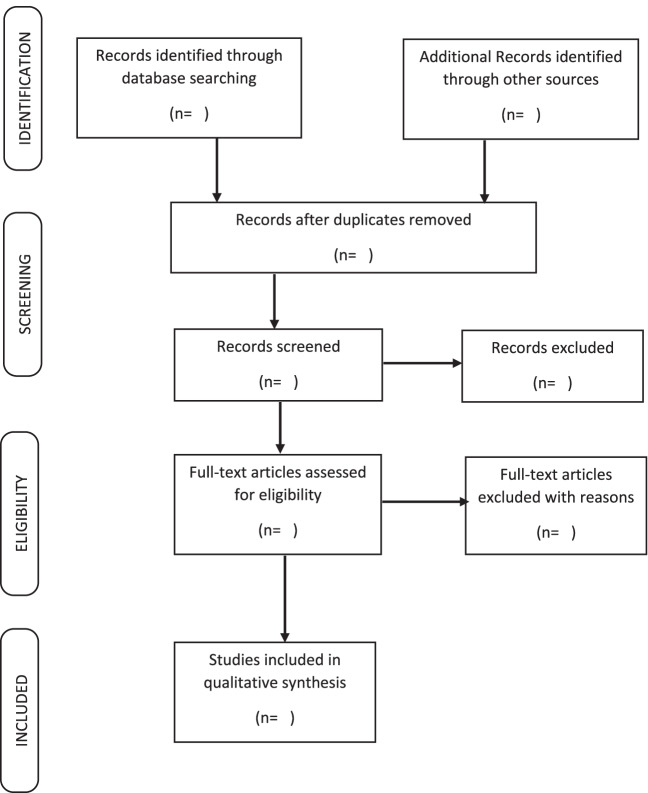
Prisma diagram for article selection process

Data will be quantitatively represented using figures and tables and qualitatively described in relation to the research question, including themes such as assessment used, similarities as well as disparate approaches, and contextual nuances. The results of the study will be reported using the Preferred Reporting Items for Systematic Reviews and Meta-analyses (PRISMA) flowchart as shown in Fig. 1.

### Quality appraisal

The Mixed Method Appraisal Tool [9] will be utilised for quality appraisal of all studies accepted after full-text review. The tool allows for appraisal of common quantitative, qualitative and mixed method studies to convey the strength of evidence from the identified studies. The MMAT uses a five-question-based appraisal tool unique to each of the study design (quantitative, qualitative or mixed methods) and scores the studies (in percentage) in 20% increasements. The higher the percentage score, the stronger the evidence. Evidence scoring below 50% would be regarded as poor quality. All scoring will be done by two members of the team to minimise systematic bias or potential errors.

## Discussion

The scoping review aims to map the assessment approaches utilised in optometry education programmes globally, in order to identify best practice approaches towards cultivating optometry graduates that share core competencies. The review will further endeavor to highlight the various factors that drive specific assessment approaches such as class size, infrastructure challenges, skills level of personnel, country or regions’ state of development, etc. The review may aid in informing schools and colleges of optometry, as well as other stakeholders such as the World Council Optometry (WCO), of assessment strategies best suited to regional or developmental contexts, whilst targeting exit level competencies aligned with the universal role of professional optometrists as primary health care providers. A further attempt will be to identify whether or not undergraduate programs in optometry across the globe share common assessment approaches. More specifically, the review will attempt to identify differences in strategies adopted to groom context-appropriate practitioners, which may be of importance when considering the strategic goals of health systems in the various countries.

An evaluation of whether the various programs utilise either summative or formative approaches, or a mixture of both, may also help to identify training programs in optometry that could be regarded as strong, based on the philosophical underpinnings of assessment approaches in higher education. Where exceptional practices with evidence-based results are found, these could serve as working models for developing schools or colleges of optometry. Considering the exit level competencies stipulated by the World Council of Optometry on what the basic job function of an optometrist should be, some coherence in assessment approaches and relevant rationale for these would be expected, if all accredited training programmes follow this global competency-based model in optometric education.

## Data Availability

Not applicable.
